# Online and Offline Prioritization of Chemicals of Interest in Suspect Screening and Non-targeted Screening with High-Resolution Mass Spectrometry

**DOI:** 10.1021/acs.analchem.3c05705

**Published:** 2024-02-21

**Authors:** Drew Szabo, Travis M. Falconer, Christine M. Fisher, Ted Heise, Allison L. Phillips, Gyorgy Vas, Antony J. Williams, Anneli Kruve

**Affiliations:** † Department of Materials and Environmental Chemistry, Stockholm University, Stockholm 106 91, Sweden; ‡ Forensic Chemistry Center, Office of Regulatory Science, Office of Regulatory Affairs, US Food and Drug Administration, Cincinnati, Ohio 45237, United States; § Center for Food Safety and Applied Nutrition, US Food and Drug Administration, College Park, Maryland 20740, United States; ∥ MED Institute Inc, West Lafayette, Indiana 47906, United States; ⊥ Center for Public Health and Environmental Assessment, US Environmental Protection Agency, Corvallis, Oregon 97333, United States; # VasAnalytical, Flemington, New Jersey 08822, United States; □ Intertek Pharmaceutical Services, Whitehouse, New Jersey 08888, United States; ● Center for Computational Toxicology and Exposure, Office of Research and Development, US Environmental Protection Agency, Durham, North Carolina 27711, United States; ◇ Department of Environmental Science, Stockholm University, Stockholm 106 91, Sweden

## Abstract

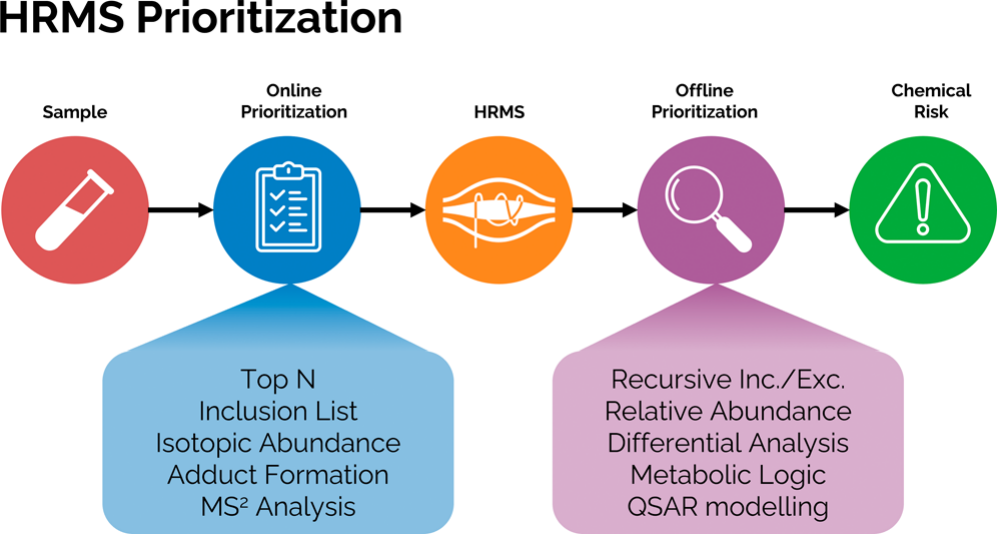

Recent advances in high-resolution mass spectrometry (HRMS) have enabled the detection of thousands of chemicals from a single sample, while computational methods have improved the identification and quantification of these chemicals in the absence of reference standards typically required in targeted analysis. However, to determine the presence of chemicals of interest that may pose an overall impact on ecological and human health, prioritization strategies must be used to effectively and efficiently highlight chemicals for further investigation. Prioritization can be based on a chemical’s physicochemical properties, structure, exposure, and toxicity, in addition to its regulatory status. This Perspective aims to provide a framework for the strategies used for chemical prioritization that can be implemented to facilitate high-quality research and communication of results. These strategies are categorized as either “online” or “offline” prioritization techniques. Online prioritization techniques trigger the isolation and fragmentation of ions from the low-energy mass spectra in real time, with user-defined parameters. Offline prioritization techniques, in contrast, highlight chemicals of interest after the data has been acquired; detected features can be filtered and ranked based on the relative abundance or the predicted structure, toxicity, and concentration imputed from the tandem mass spectrum (MS^2^). Here we provide an overview of these prioritization techniques and how they have been successfully implemented and reported in the literature to find chemicals of elevated risk to human and ecological environments. A complete list of software and tools is available from https://nontargetedanalysis.org/.

## Introduction

The scale and impact of novel entities, such as persistent organic pollutants (POPs), toward the sustainability of the planet’s ecosystem are poorly understood.^1^ Domains contributing to the impact on this planetary boundary include industrial manufacturing,^2^ the supply and quality of foods,^3^ and finished pharmaceutical products.^4^ The Chemical Abstracts Service (CAS, American Chemical Society) currently reports >200 million organic substances, alloys, coordination compounds, minerals, mixtures, polymers, and salts.^5^ Global production of many chemicals is increasing each year, exceeding our ability to assess the risk posed by these chemicals, which may lead to adverse impacts on the environment.^2,6^ Methods utilizing analytical chemistry tools, such as high-resolution mass spectrometry (HRMS), are constantly being improved upon to detect and measure the mass of a wide range of chemicals with extreme precision. However, the amenability of a chemical to a specific analytical method depends on many factors, including the extraction methodology, chromatographic conditions, instrument response factor, and the concentration of the chemicals present in the sample (*vide infra*).^7^

A variety of HRMS instruments are used to detect chemicals in a sample where the mass-to-charge ratio (*m*/*z*) of each chemical is measured with a mass resolution >10,000.^8^ The instruments most often used are time-of-flight (TOF) and orbital ion trap mass analyzers coupled with quadrupole devices to enable ion isolation (*m*/*z* window widths between 0.4 to 2 Da) and/or dissociation for multidimensional data acquisition.^9^ With most data-dependent acquisition (DDA) methods, precursor ions are isolated and fragmented using molecular dissociation, where the resulting fragments are analyzed with a high-resolution mass analyzer. Unfortunately, the number of spectra per unit time is limited such that DDA does not enable MS^2^ analysis of all precursor ions; therefore, the MS^2^ spectra must be triggered based on certain criteria. Alternatively, data-independent acquisition (DIA) methods set the quadrupole to pass through all ions or ions in a wide *m*/*z* range (typically a few tens of Da), to the collision cell for dissociation. In DIA, the resulting MS^2^ spectrum contains a mixture of fragment ions from all precursors in the MS^1^ spectrum. There are two strategies used to deconvolute the precursor and fragment mass spectrum: post hoc analysis of the chromatographic peak profile^10^ and multiway curve resolution. The former has been thoroughly tested and used, but it can produce lower-quality MS^2^ spectra when presented with numerous coeluting precursors.^11^ The latter is an emerging technique that has been demonstrated to improve deconvolution by adding the analysis of the relative peak intensity to the data cube.^12^ Ion mobility separation is a complementary technique that can be coupled to HRMS instruments to separate coeluting chemicals and deconvolute MS^2^ spectra based on their collisional cross sections (CCSs).^13^

The two main approaches for high-throughput detection and identification of unknown chemicals using HRMS are known as suspect screening and non-targeted screening^14^ (also non-targeted analysis). Generally, suspect screening matches the acquisition data with a list of chemicals of interest in the sample, thereby ignoring any other chemicals which may be in the sample. On the other hand, non-targeted screening does not require *a priori* assumptions for the presence or absence of chemicals in a sample, which often results in a wealth of information-rich data that can be more challenging to process efficiently. Careful design of suspect and non-targeted screening workflows is crucial for the successful detection and identification of chemicals present in samples. The large number of features that can be detected, even in seemingly “clean” matrices, illustrates the complexity of non-targeted data. For example, in previous studies of both tap water and tertiary-treated wastewater, tens of thousands of features were detected across multiple samples.^15,16^ Here, “feature” refers to a data tuple consisting of the retention time of the chromatographic peak and *m*/*z* of the precursor adducts and isotopes.^17^ The confident structural annotation of unknown features has been a topic of great interest, with molecular networking,^18,19^ forward prediction,^20,21^ and inverse prediction^22,23^ technologies recently becoming available. The confidence of annotation performance can be quantified for communication of results.^24,25^ Regardless of the selected workflow, a considerable amount of computational and research time is currently required to annotate each feature confidently. Therefore, there is a need to develop strategies that will reduce analysis time without sacrificing confidence.

Prioritization strategies address this need by highlighting the most relevant features to the study goal, which can significantly reduce the vast number of features detected by HRMS that are selected for further investigation. The field is working to standardize the evaluation and performance of suspect and non-targeted screening workflows,^26−28^ and prioritization strategies are playing an important role in these workflows. Here, we provide a framework for the categorization of online and offline prioritization techniques and methodologies for the harmonization of prioritization strategies by demonstrating concepts and tools available to researchers that can facilitate both high-quality HRMS analysis and communication of the results, including confidence in those results.

## Prioritization Lists

Lists of various chemicals can be employed in both online and offline prioritization strategies to narrow down potential suspects. Both chemical databases and mass spectral libraries can be used to curate a “prioritization list”. While the former contains useful information for each chemical, such as its structure, names, physicochemical properties, functional uses, and toxicity,^29^ mass spectral libraries contain empirical MS^1^ and/or MS^*n*^ spectra for each chemical that can be used as a direct comparison with experimental data for identification^30^ (see Suspect Screening). Studies have used hundreds,^31^ thousands,^14^ or tens of thousands of chemicals^32^ relevant to the scope of the analysis; however, in most cases, it would be antithetical to the aim of prioritization to report hundreds or thousands of chemicals based on matching exact masses with a list or database without further context. Therefore, building a list of suspect chemicals for online or offline prioritization is of utmost importance and should be clearly described and reported. Depending on the scope of a particular study, chemical lists based on their structural, property, and regulatory information may be used to more effectively prioritize chemicals of interest for further investigation and provide clear context in the reporting.

**Figure 1:**
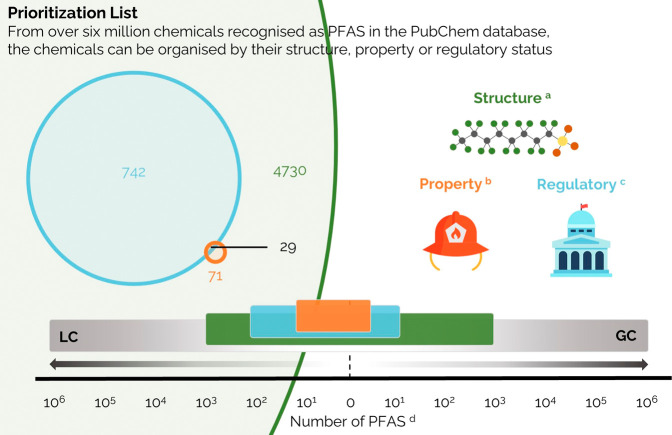
An example of the structural, property, and regulation-based characterization for PFAS in the LC- and GC-amenable chemical spaces. From over 6 million chemicals defined as PFAS,^33^^*a*^4730 were identified by the OECD,^34^^*b*^at least 71 chemicals have been identified in aqueous-film-forming-foam (AFFF),^35^ and ^*c*^742 are listed as REACH chemicals (EU-1272/2008). Source: NORMAN Suspect List Exchange.^36^^*d*^Chromatography predictions were calculated by Alygizakis et al.^37^ and extrapolated to the entire PubChem list.

Structure-based lists incorporate sets of chemicals of similar functional groups, moieties, or the presence of specific elements. For example, the detection of organic pollutants in the environment can include common structure-based lists including polychlorinated biphenyls (PCBs), polybrominated diphenyl ethers (PBDE), and per- and polyfluoroalkyl substances (PFAS).^38,39^ Incidentally, each of these three groups of chemicals may be assembled into a larger organohalogen compounds (OHCs) list. While PCBs and PBDEs are commonly separated by gas chromatography (GC), PFAS from the Organisation for Economic Co-operation and Development (OECD) priority list are more often LC-amenable (Figure 1). Due to the influence of physicochemical properties and amenability to specific techniques, the selection of chemicals for specific structure-based lists will depend on instrument selection and sample preparation.^7^ Additionally, the toxicity or hazard of chemicals with similar structural and molecular descriptors can be predetermined and used to rank chemicals based on their predicted risk to human and ecological health, as demonstrated in a proof-of-concept software application developed by the United States Environmental Protection Agency (US-EPA).^40^

Property-based lists may incorporate chemicals that share similar physicochemical parameters or are used to elicit the same desired outcome. For example, herbicides, fungicides, insecticides, or rodenticides, collectively known as pesticides, are property-based lists because they all have the same aim: to cause adverse effects in their target organism.^41^ Similarly, pharmaceuticals and personal care products (PPCPs) are another property-based group of chemicals that are all designed to improve the quality of life. Property-based lists may also include chemicals with similar physicochemical properties or environmental fate, such as transformation products from pesticides or pharmaceuticals, which can be included to monitor drinking water quality before and after treatment.^42^ Such lists are readily available from resources such as the NORMAN Suspect List Exchange^36^ and the US-EPA’s CompTox Chemicals Dashboard.^43^

Regulatory-based lists of chemicals are prepared by local, state, or federal governments to limit the exposure of chemicals with known adverse impacts to human, societal, or ecological health. For example, the Registration, Evaluation, Authorisation and Restriction of Chemicals (REACH; EU/1907/2006) and the US-EPA’s Toxic Substance Control Act (TSCA; 15 U.S.C. §2601 1976) each contain a list of substances that have been used to screen for restricted chemicals in textiles and surface waters.^44,45^ Similarly, lists of controlled or illicit substances can be used to extend the capability of forensic laboratories,^46^ for example, the Scientific Working Group for the Analysis of Seized Drugs (SWGDRUG), which publishes structure-based lists and mass spectral libraries.^47^ Typically, it takes many years of scientific reporting for a chemical to appear in regulatory-based lists and is subject to political processes.^48,49^ Many of these chemicals are still manufactured and used within regulatory frameworks, often to balance the adverse effects on humans and the environment with the economic value of the chemical. For these reasons, regulatory-based lists are a popular starting point for many studies, as the detection and quantification of these chemicals provide key monitoring information to enforcement agencies.^50^ It should be noted that transformation products of regulated chemicals, resulting from biological metabolism and environmental degradation, should also be taken into account (Structural and Molecular Analysis).

## Online Prioritization

**Figure 2:**
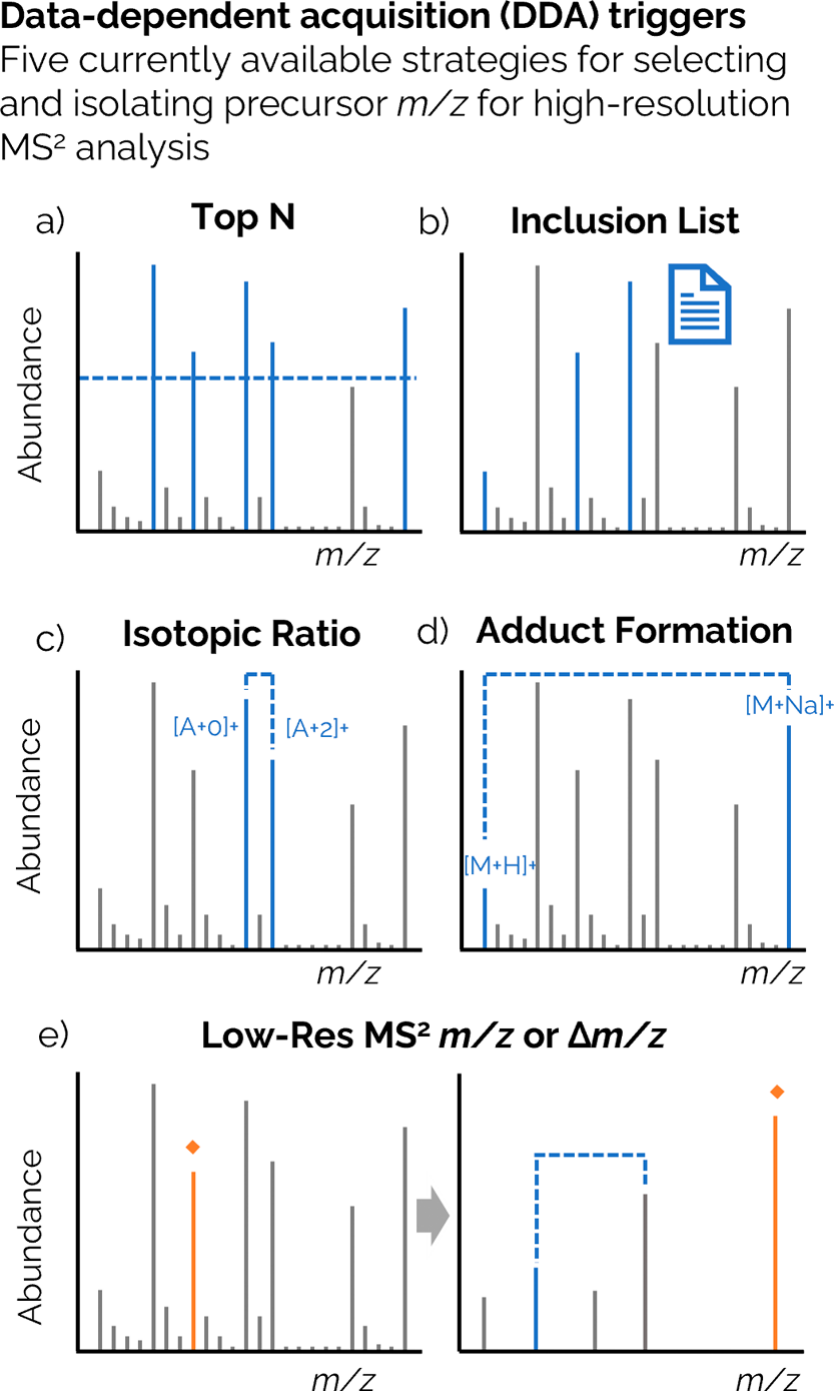
Examples of current online prioritization strategies for the selection and isolation of precursor *m*/*z* for fragmentation: (a) Top *N*, (b) inclusion list. Then, (c) isotopic ratio, (d) adduct formation, and (e) low-resolution MS^2^ signal processing are currently limited to a few instruments.

Online prioritization includes the variety of parameters that are used to perform DDA, predefined by the user for automatic selection by an instrument in real-time during each duty cycle. The five online prioritization techniques that are used to overcome the duty cycle limitations in DDA are (1) Top *N*, (2) Inclusion/Exclusion List, (3) Isotopic Abundance, (4) Adduct Formation, and (5) Low Res MS^2^ Trigger. The simplest and most widely available strategy is the “Top *N*” selection (Figure 2a), where *N* most abundant MS^1^ peaks from a single scan, above a set intensity threshold, are selected for MS^2^ analysis.^42,51−53^ Most HRMS instruments also allow triggering MS^2^ experiments via matching with an inclusion list of predefined *m*/*z* values (Figure 2b),^44,54,55^ which may be built from a prioritization list as described in Prioritization Lists.

A useful approach for the curation of the inclusion list is a post hoc analysis of MS^1^ data previously acquired. Here, precursor *m*/*z* values can be matched with a suspect screening prioritization list to identify potential candidates. Due to the lack of relative confidence in matching only MS^1^ data and limitations of duty cycle, these candidates can be fed back into an inclusion list, where each of the candidates can be isolated for fragmentation and analyzed to obtain a higher degree of annotation confidence and to target more ions for MS/MS. Conversely, features previously triggered for MS^2^ acquisition can be added to an exclusion list, allowing other peaks to be interrogated in subsequent runs.^56^ These approaches require multiple injections and data analysis that will increase the total user time for acquisition. However, pooled quality control samples can be used to generate an inclusion list without requiring multiple injections of every sample.^57^

Triggering strategies can also involve the real-time determination of the isotopic abundance pattern; for example, chemicals containing Cl and Br can be triggered for MS^2^ based on the increased intensity of the [A + 2]^+^ isotope caused by their naturally occurring stable isotopes (Figure 2c). Similarly, the presence of adduct formations ([M + H]^+^, [M + Na]^+^, [M + K]^+^, etc.) can be determined and selected for fragmentation accordingly (Figure 2d).^58^ Finally, in instruments with two or more mass analyzers, the *m*/*z* or Δ*m*/*z* from a rapid (40 Hz) and low-resolution (>0.1 Da) MS^2^ spectrum can be monitored for peaks of interest.^59^ This can, in turn, trigger a high-resolution MS^2^ experiment in the next cycle (Figure 2e). Triggering MS^2^ spectra based on the measured isotopic ratio, or the presence of adducts in the MS^1^ scan is, to the authors’ knowledge, available only for the instruments from one vendor (Thermo Fisher Scientific) and has not been widely employed for the analysis of environmental contaminants. However, these strategies were recently used to prioritize the presence of potentially harmful chemicals in water by triggering MS^2^ acquisition based on the presence of halogen isotopic ratios in the MS^1^ and by neutral losses associated with structural alerts.^58^ These approaches appear to be suitable for the detection of small molecules though they have not yet been extensively tested, optimized, and validated for applications to suspect and non-targeted screening approaches.

Each of these approaches aims to select chemicals of interest for rapid and efficient fragmentation but have certain limitations. First, chemicals of equal concentrations can have differences in response factors by several orders of magnitude.^60^ Particularly in complex matrices like foods and biosolids, chemicals of interest may yield low signal intensity due to low ionization efficiency or matrix effects; therefore, the MS^2^ spectra may not be acquired for these ions with “Top *N*”. Finally, as mentioned earlier, only a limited number of *m*/*z* values matched to an inclusion list may be isolated depending on the size of the inclusion list and the extent of coelution of these compounds. Certain strategies can be used to address some of these limitations, that help optimize the selection criteria of MS^2^ acquisitions.

The chromatographic method also plays an important role in determining the extent of coelution and can limit the number of precursor ions that can be triggered for MS^2^ acquisition.^61^ Shorter gradients typically yield sharper, more intense peaks, while longer gradients often increase peak widths and have reduced intensities. Longer gradient elutions also enable the detection and fragmentation of more precursor ions in highly complex samples by improving the overall chromatographic peak resolution.^62^ These effects can contribute to the efficiency of the instrument to select and isolate peaks within the duty cycle time of the instrument for reliable analysis. As a result, 20–30 min gradient lengths have been found to provide a good balance between peak resolution and separation, cycle time, and total run time.^63−65^ Additionally, “active” or “dynamic” exclusion windows can avoid overtriggering multiple MS^2^ spectra for the same precursor so those scans can be used to generate MS^2^ spectra for more unique precursors. The duration of these exclusion windows should be set based on general peak widths of the chromatographic method to ensure that MS^2^ spectra are collected at or near the apex of a peak (i.e., for a 10 s peak, the exclusion window should be <5 s).

In DDA which is applied by using an inclusion list, instruments from many vendors also allow for the selection of chemicals within a specified retention time window to increase the number of chemicals monitored in a given duty cycle. If the retention time is unknown or not measured with analytical standards, the retention time for any chemical may be predicted using advanced machine learning models.^66−68^ Here, the calculated or predicted retention time index (RTI) for each chemical in the inclusion list can be used to assign an appropriate retention time window. Additionally, employing machine learning algorithms to predict the polarity and LC amenability can be used to filter inclusion lists.^37,69^ To the authors’ knowledge, this process has yet to be applied to explicitly filter chemicals in inclusion lists for DDA but is expected to improve prioritization given sufficiently accurate models for predicting retention time.^70^

Improvements in hardware continually improve signal quality at lower cycle times, and developments in real-time signal processing will allow more useful and dynamic background exclusion and inclusion. Given the current limitations of DDA on chemical selection for fragmentation, DIA provides an alternative instrumental technique to improve MS^2^ coverage,^71^ although because it lacks the ability to isolate precursors, it is not amenable to online prioritization techniques. Deconvolution of DIA data requires complex post hoc signal processing to link MS^2^ spectra with corresponding MS^1^ peaks. Retention time correlation models can provide adequate deconvolution results,^72^ and developing multivariate curve resolution analysis can refine the signal processing even further.^73^

## Offline Prioritization

### Suspect Screening

Offline prioritization strategies employ post hoc analysis of MS^1^ and MS^2^ (both DIA and DDA) data. Regardless of the acquisition methodology, offline prioritization ranges in complexity from suspect screening to non-targeted screening, each of which are performed independently of each other. Data processing in suspect screening begins by mining the data directly for acquired MS^1^ features with the exact mass and isotopic distribution in a prioritization list (see Prioritization Lists). Measured and/or predicted retention times help to filter the candidates if multiple matches are observed.^66^ Substances with unique mass defects or repeating moieties, such as PFAS, can be extracted and prioritized in suspect screening workflows.^74,75^ Finally, matching the MS^2^ spectra with a publicly available spectral library or an in-house library increases the confidence of a positive match.^25^ Inventories that detail the volume or concentration of the discharge of chemicals to the environment have been used to prioritize substances emitted from a wastewater treatment plant.^76^ The advantage of this strategy is the ability to assess the risk of exposure to downstream environments, based on the relative discharge rates declared by licensed companies.

Prioritization lists containing thousands of chemicals may be used in offline prioritization; however, the propensity for false positives increases with the length of the prioritization list. Hence, it is beneficial to reduce these lists where possible depending on study questions and goals. Specifically, caution is urged when using complete PubChem and ChemSpider databases containing over 100 million live chemicals from 799 and 277 sources, respectively.^77,78^ The probability of most of these chemicals being detected in their respective matrix is extremely small, so an effort has been made to limit the chemical list used to support mass spectrometry analysis to a much smaller fraction of the database: the so-called PubChemLite for Exposomics list that is updated regularly.^79^

### Intensity and Concentration Exclusion

**Figure 3:**
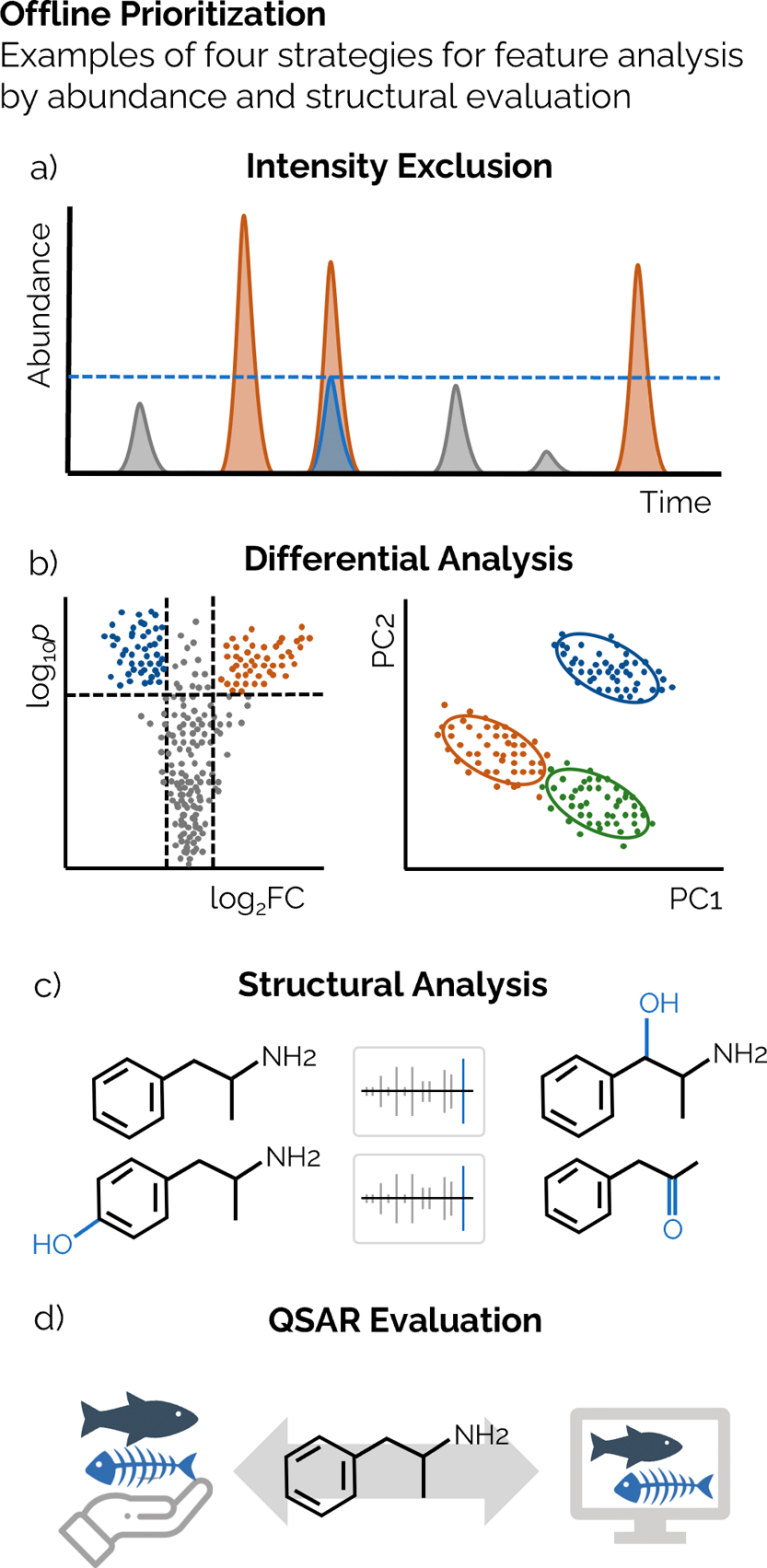
Examples of offline prioritization techniques used to highlight or rank features detected by HRMS. (a) Peaks with lower response than a spiked chemical are excluded from analysis. (b) Fold-change analysis and principal component analysis reveal grouped features. (c) Metabolic logic and analysis of MS2 spectra group features of similar structure. (d) Empirical or predicted QSAR rank features based on biological endpoints.

A strategy common for the non-targeted screening of impurities in food, pharmaceuticals, and medical devices involves filtering features based on the instrument response relative to an internal standard of known concentration (Figure 3a).^80,81^ Threshold of toxicological concern (TTC), also known as safety concern threshold (SCT), analytical evaluation threshold (AET), or dose-based threshold (DBT), are offline prioritization strategies developed to highlight chemicals by excluding those expected to have low concentrations or risk of adverse effects.^82^ This approach has been used to prioritize the presence of chemical migrants in food that originated from food contact material^83,84^ and potentially harmful chemicals detected in natural and drinking water.^85^ This strategy was also demonstrated for the analysis of extractables and leachables (E&L) from a coronary implant, where polymer-based chemicals and transformation products were positively identified.^86^

There are notable exceptions to the TTC approach, where chemicals containing known structural alerts should be evaluated regardless of their intensity or concentration, commonly known as the “cohort of concern”.^82,87^ However, in an NTS approach, the absence of *a priori* structural information can obfuscate confident annotation of chemicals with these structural alerts and may be omitted from the analysis. Furthermore, poor quantification accuracy often results when using surrogate internal standards^88^ due to variability in the ionization efficiency between chemicals.^60^ For example, bifenthrin (CASRN: 82657-04-3), a potent insecticide and aquatic toxicant, has a relatively low ionization efficiency (logIE: −1.19 to 0.79),^89^ where its concentration could be seriously underestimated using standard practices.^82^

### Differential Analysis

Differential analysis relates to a number of methodologies that aim to help isolate features of interest, without a predefined list of chemicals, based on the analysis of multidimensional data within a sample, and the statistical differences between and within samples (Figure 3b). These types of analysis determine differences in feature presence and intensity between any two (or more) groups (temporal, spatial, biological, etc.) and are a valuable approach to identify chemicals of interest in a particular sample group. For example, the chemical diversity from different brands and collection dates of oats were compared using principal component analysis (PCA), where even this relatively simple food-type resulted in >3000 unique features. This work concluded that differential analysis was an efficient prioritization approach for highlighting QCs in a spiked vs unspiked sample, but that the best approach depends on the goal and may require a combination of multiple approaches.^90^ This type of trend analysis has also been useful in the analysis of human blood,^55^ drinking water,^91^ food,^92^ biological matrices,^93^ contaminated beverages,^94^ and human biomonitoring.^95^

As data acquired with HRMS is highly dimensional and can contain many more variables and observations, univariate or multivariate statistical tests can be scaled up by applying supervised and unsupervised machine learning algorithms to build predictive models that resolve complex relationships in the data. For example, advanced trend analysis has been applied to characterize and prioritize features that were detected in wastewater-impacted surface waters, where 25 contaminants were successfully identified.^96^ These features could also be categorized into periodic, spill, increasing, or decreasing trends that can enable more efficient filtering and chemical risk assessment. The accuracy and precision of these predictive models continue to be developed and evaluated toward the high-throughput and multiresidue analysis of chemicals from samples.^97^ In any case, the selection and purpose of the statistical methodologies should be reported. Futhermore, since the number of features generated by HRMS is often much greater than the number of samples or observations, the application and interpretation of statistical analysis should be verified and carefully considered to avoid overfitting.^75^

### Structural and Molecular Analysis

Prioritization of features found in samples can be conducted by grouping potential transformation products (metabolic logic).^98^ This analysis is performed first by identifying features with a mass difference equal to known biotransformation reactions, including basic modifications, conjugation, and deconjugation (Figure 3c).^99^ As many basic modifications are more polar than the parent compound, a simple filter can be applied to features with longer retention times in reverse-phase LC. Then, the similarity of MS^2^ of the parent and potential transformation products can be evaluated, where the fragments of transformation products should generally align with the parent chemical. This strategy was partially successful in the prioritization of features detected in treated wastewater^100^ and later more successful in prioritizing pharmaceutical transformation products in sludge.^101^ Tools commonly used for the prioritization and identification of transformation products include BioTransformer,^102^ enviPath,^103^ and the US-EPA’s Chemical Transformation Simulator (CTS).^104^

Molecular networking can be used to prioritize features based on expected structural similarities determined by comparing high-quality MS^2^ data between features.^105^ Similar to metabolic logic, this analysis can reveal chemicals of analogous structure or chemicals with similar bioactive chemicals and metabolites. Molecular networking is a well-suited application for the dereplication of novel pharmaceuticals, especially from existing natural products.^106^ Frameworks are available to freely communicate these types of analysis and often include powerful visualization tools.^18,107^ Molecular networking was used to help identify toxic alkaloids from a case of poisoning from an unidentified plant root, where chemical profiling helped to identify the genus and species of the plant that produced the toxins.^108^

The negative mass defect of fluorine and identification of homologous series can be leveraged to prioritize legacy and emerging PFAS. Specific tools have been developed to detect differences in mass defect,^109^ with the ability to discern PFAS by evaluating the relationship between mass defect (MD) and mass (m) relative to the number of carbons (C) (MD/C – m/C).^110^ These analyses has been applied to the discovery of novel PFAS in historical pine-needle samples by the combination of the negative mass defect and characteristically low CCS values.^111^

### Quantitative Structure–Activity Relationship Evaluation

Hazard profile listings for chemicals contain various types of empirical human and ecological toxicity endpoints, including acute and chronic, reproductive, and behavioral, from oral, inhalation, and dermal exposure pathways.^112^ Chemicals from these listings can be matched with tentative structural annotation from suspect and non-targeted screening workflows to evaluate the potential chemical risk. When these hazard profiles are paired with cheminformatic data, quantitative structure–activity relationship (QSAR) models can be developed to provide hazard estimates from chemicals with no empirical evidence (Figure 3d). QSAR-based strategies for offline prioritization aim to rank a list of putative identifications according to their associated risk to receiving organisms and environments.^113^ QSAR models are typically determined by the relationship between a chemical’s structural and/or molecular descriptors and its human or ecological-based toxicity endpoint. These models can range in complexity; for example, bioaccumulation in aquatic organisms can simply be related to a chemical’s octanol–water coefficient (log *K*_ow_),^114^ and toxicity can be predicted by analyzing key substructures that are known to negatively impact survivability and reproduction.^115^

Human and ecological toxicity predictions can also be used to prioritize hazardous substances from a list of features. Analysis of the relative abundance of chemicals detected in household dust (via ToxPi^116^) revealed 15 compounds that were reported in dust samples for the first time.^117^ However, toxicity predictions that are derived from structural information^118^ or the presence of structural alerts^58^ contain two sources of uncertainty that can compound the error of the estimate: the putative structural annotation and the toxicity prediction model.^25^ To address this issue, Peets et al.^119^ modeled acute aquatic toxicity in fish-derived only from the MS^2^ spectrum (MS2Tox), precluding confident annotation. Additionally, there have been recent advances to quantify chemicals identified by non-targeted screening through machine learning algorithms, enabling more accurate evaluation of the risk posed to organisms exposed to contaminated environments.^88,113,120^

## Conclusion

For suspect or non-targeted screening methodologies to be most useful for the evaluation of chemical risk to human or ecological health, the chemical space and prioritization strategies must be clearly defined. Careful consideration of the data acquisition and data analysis parameters presented in this study can vastly improve the confidence and efficiency for the prioritization of chemicals of interest in environmental, biological, food, medical device, and other matrices. We encourage HRMS users employing suspect and non-targeted screening workflows to conceptualise prioritization strategies in the framework presented here. Online prioritization techniques require minimal input from the user and can enable the generation of high-quality MS^2^ spectra for a greater percentage of relevant features depending on the study goals. Through the use of one or more offline prioritization techniques, both DIA and DDA information can be used to highlight chemicals based on their abundance, chemometrics, or predicted impacts to a selection of toxicological endpoints. Notable implementations of each of these strategies have been presented in this study, and we support the continued development of each of these strategies as the field matures.
